# Assessment of biosafety and toxicity of hydrophilic gel for implantation in experimental in vitro and in vivo models

**DOI:** 10.1186/s40360-022-00577-3

**Published:** 2022-06-08

**Authors:** N. Bezdieniezhnykh, A. Lykhova, T. Kozak, T. Zadvornyi, T. Borikun, O. Voronina, N. Lukianova

**Affiliations:** grid.418751.e 0000 0004 0385 8977 RE. Kavetsky Institute of Experimental Pathology, Oncology and Radiobiology, NAS of Ukraine, Kyiv, Ukraine

**Keywords:** Hydrophilic gel for implantation, Experimental model systems, Toxicity, Genotoxicity, In vitro and in vivo studies

## Abstract

**Background:**

The assessment of biosafety of pharmacologically active substances is crucial for determining the feasibility of their medical use. There are controversial issues regarding the use of substances of different origins as implants.

**Methods:**

We have conducted the comprehensive studies to determine the in vivo toxicity and in vitro genotoxicity of new generation of hydrophilic gel for implantation (production name of the substance “Activegel”) to detail its characteristics and assess its biosafety.

**Results:**

In vivo studies have shown the absence of clinical manifestations of intoxication in animals and no abnormalities in their physiological condition, general and biochemical blood tests. Evaluation of the site of the gel application showed no inflammatory reaction and evidenced on normal state of tissues of animal skin. The results of the genotoxicity test indicated that the gel did not affect the parameters of DNA comets and the formation of micronuclei, accordingly, had no genotoxic effect on human peripheral blood lymphocytes. When studying the effect of the gel on malignantly transformed cells in vitro, it was found that the gel for implantation did not change the proliferative activity and viability of human breast cancer cells.

**Conclusions:**

Comprehensive in vitro and in vivo study using various experimental model systems showed that the hydrophilic gel for implantation “Activegel” is non-toxic.

**Supplementary Information:**

The online version contains supplementary material available at 10.1186/s40360-022-00577-3.

It is known that toxicological research is now mandatory among the most important steps in the biological assessment of potential drugs and medical devices, as it allows to identify possible adverse effects of the test substance on the body and prevent side effects. Such studies include both in vitro experiments and in vivo toxicity testing [1, 2]. Due to such a comprehensive approach the use of a new pharmacological substance in clinical practice is justified, the doses are adequate, and the mode of application is safe. In particular, the aim of preclinical toxicological studies of a pharmacological substance in the in vivo system is to establish the nature and severity of its potential harmful effects on the body of experimental animals and to assess its safety [3].

Therefore, it is extremely important to control the means used for implantation, because today there is a lot of controversial problematic issues regarding the use of such substances and their safety in humans [4]. Such substances are used not only for implantation to compensate for soft tissue insufficiency, to increase the volume of the mammary glands, correction of soft tissues due to aging, but also to eliminate certain postoperative and post-traumatic soft tissue deformities, and scars due to injury. It is especially important to control the substances used in cancer patients, in particular after surgery to correct defects [5, 6].

In the treatment of cancer patients, one of the most common and important methods is chemotherapy, but there are many problems regarding the use of many antitumor agents, including their hydrophobicity, low bioavailability, instability, high toxicity, severe side effects, and lack of targeted action [7–9]. Therefore, an important area is the development and evaluation of new delivery systems for anticancer drugs for the treatment of tumors with higher efficiency and lower toxicity. In particular, local application of cancer drugs could minimize their toxic effects on normal tissues with maximum effectiveness against malignantly transformed cells [10–12]. The hydrogel-based drug delivery system is currently attracting more and more attention. Due to its liquid structure, hydrogel could be easyly combined with medicinal preparations for subsequent targeted delivery [13].

The universality of hydrogels is determined by their structure, which could be technically regulated for the purposes of invention of new polymers, new approaches to their crosslinking and new strategies for their production [14]. But it should be understood that such new approaches to translational biomedicine in vivo as the use of polymers as vectors for the delivery of bioactive molecules require proper control of their toxicity and safety for the human body [15].

In this work, we have investigated a synthetic polymer (2–4%) - new generation hydrophilic gel with the production name “Activegel”. In particular, we have evaluated in vivo toxicity of hydrogel using various experimental model systems and its effect on tumor cells in vitro to understand the possible consequences of its potential use as an implant material or further expansion of its usage in clinical practice, in particular, as a vector for targeted delivery of anticancer preparations.

## Materials and methods

### Test substance

Sterile medical device “Activegel” is a sterile 2–4% mixture of synthetic polymer (new generation hydrophilic gel, produced by LLC “National Center for Medical Technology”, Ukraine; packaging: Container PP (MAGIFLEX BAG PVC-free) with a volume of 100 ml), designed for use after invasive surgical procedures in order to obtain long-term results.

### In vivo study

The study was designed to achieve the objectives of the experiment using the minimum number of animals. All experiments were conducted in accordance with bioethics standards and permission from the Commission on Bioethics of RE Kavetsky IEPOR of NAS of Ukraine. Wistar rats of both sexes 2–2.5 months old (males weighing 170–210 g, and females weighing 150–180 g) were used. The conditions for keeping the animals met the standards specified in the manual The Guide for Care and Use of Laboratory Animals (ILAR publication, 1996, National Academy Press, 1996).

The main study was conducted with 4 animals of each sex in the appropriate group. Animals in the experimental groups were distributed as follows: I stage. Data analysis within 48 hours after Activegel injection. The total number of rats within the stage - 16 males and 16 females (total - 32 animals). Stage II. Data analysis within 14 days after Activegel injection. The total number of rats within the stage - 16 males and 16 females (total - 32 animals). Subcutaneous injection of the substance: 48 hours before the administration of the test substance, the area of ​​hair on the rat’s back (3 cm × 3 cm) was trimmed using a universal trimmer PHILIPS Multigroom series 7000 MG7735 / 15. The substance was administered subcutaneously with a Bog Mark microinjection syringe (Poland) and a 20 ml syringe needle (Momina Krepost, Bulgaria). The injection site was pre-treated with 96% ethanol.

In order to study the acute toxicity of the medical product “Activegel”, the substance was administered to experimental animals subcutaneously (s.c.). The animals of each sex were distributed in four groups: 3 experimental and 1 control. The results were analyzed 48 hours and 14 days after administration of the test substance. In the study 3 doses of the product “Activegel” were used (according to ENV/JM/MONO(2016)32 No. 237 “Guidance document on considerations for waiving or bridging of mammalian acute toxicity tests”): 1) low - 500 mg/kg; 2) medium - 2000 mg/kg; 3) high - 5000 mg/kg. Animals of the control group received s.c.administration of saline solution (Yuriya-Pharm, Ukraine, S. AA8175/1–1) in a volume corresponding to the highest of the tested doses of the preparation.

Forty-eight h and 14 days after injection of “Activegel”, the animals were humanely killed by overdose by inhalation anesthesia, and subsequent studies (macroscopic and cytomorphological examination of organs and tissues, general and biochemical blood tests) were performed.

#### Macroscopic study of the internal organs of experimental animals

A macroscopic analysis of the condition of rat skin tissues at the location of the “Activegel” application, as well as the state of the internal organs of rats (brain, heart, lungs, liver, kidneys, thymus, spleen, testicles and appendages, uterus, ovaries and appendages) was conducted. The size of the organs, their color, shape and weight were evaluated.

#### General and biochemical analysis of peripheral blood of experimental animals

Blood samples were drawn from the retroorbital venous sinus, and used for general and biochemical analysis. The cellular composition of peripheral blood (erythrocytes, leukocytes, band neutrophils, segmented neutrophils, lymphocytes, monocytes, eosinophils), hemoglobin, erythrocyte sedimentation rate (ESR), as well as biochemical blood serum indices (concentration of total bilirubin, calcium, creatinine, glucose, total protein, urea, C-reactive protein, inorganic phosphorus, as well as the activity of ALT and AST) were quantified on an automatic biochemical analyzer ChemWell 2310 according to the instructions of the manufacturer of the device and the corresponding reagents and using software for GBG ChemWell®. Quality control of laboratory tests was tested using control serum with known normal and abnormal values of these indicators (control serum level 1 and 2 Global Scientific). The control values were within the set range.

#### Morphological examination of rat skin tissues from the injection site

To carry out cytomorphological analysis, skin, muscle and subcutaneous fat at the location of “Activegel” application were removed and analyzed. Tissue samples were fixed in 10% neutral buffered formalin solution. After standard histological conduction in alcohols of increasing concentration, the material was poured into paraffin. Sections 4–5 μm thick were made from paraffin blocks and stained with hematoxylin and eosin.

### In vitro experiments

#### Evaluation of the hydrophilic gel effect on the viability and proliferation of malignant cells

##### Preparation of the test substance samples

Samples of the hydrophilic gel were added to the wells of a 12-well plate (0.4 g/well) in 3 repetitions. Physiological saline was used as a negative control (0.4 g/well). Next, 2 ml of complete DMEM medium with 10% FCS were added to wells with hydrophilic gel or physiological saline, and the plate was incubated in the presence of 5% CO_2_ at 37 °C and 100% relative humidity for 48–96 hours.

After 48 and 96 hours of incubation, the gel was separated from the medium by centrifugation (1000 rpm 5 minutes). Then the supernatant (conditioned media) from each sample were collected and stored at + 4 °C.

##### Cell сultivation

Human breast cancer cells of MCF-7 and MDA-MB-231 lines were obtained from the Bank of Cell Lines from Human and Animal Tissues of the RE Kavetsky IEPOR NAS of Ukraine. The cells were cultured in complete DMEM medium with 10% fetal calf serum (FCS) in a humidified atmosphere with 5% CO_2_ at 37 °C. Replacement of the medium and cell passaging were performed according to standard methods. The cells were passaged after reaching 80% confluence at a density of 10^5^ cells/ml using Versene solution.

##### Determination of cell viability by colorimetric method

Cells of MCF-7 and MDA-MB-231 lines were seeded in wells of a 96-well plate in DMEM medium with 10% FCS (1 × 10^5^ cells/ml) in a volume of 150 μl/well and cultured for 24 hours. Then the conditioned media were added to the appropriate wells of a 96-well plate (150 μl) in different ratios: 100, 50, 25, 12.5, 6.3, 3.1, 1.6% (at least three replicates per each concentration). The conditioned media of the cells cultivated after addition of saline solution served as negative control. Then the cells were cultured for another 72 hours, and the number of living cells was assessed visually (direct microscopy), and using colorimetric method by staining the cells with crystal violet [16]. The results were recorded using a spectrophotometer (Labsystems Multiskan PLUS, Finland) with a vertical beam path at an excitation wavelength of 540 nm.

The number of living cells (X1) in each well of the test sample, as a percentage, was calculated by the formula:

$${X}_1=\frac{{\mathrm{A}}_1\cdot 100\%}{A_0},$$

where A_0_ is the average value of the optical density in the control wells; A_1_ is the average value of the optical density in the wells with the test sample.

The average number of living cells for each test sample dilution was calculated.

##### Evaluation of in vitro genotoxicity

The study was performed in vitro on a model of human peripheral blood lymphocytes. Peripheral blood samples (9 ml) were taken from 4 healthy donors (were informed about the study and gave written consent to the use of biological material for research purposes) (2 males aged 29 and 45 years, and 2 females aged 29 and 33 years).

Lymphocytes were isolated from whole peripheral blood using the method of Boyum [17]. Blood twice diluted with saline was layered on a solution of Histopaque in a ratio of 3:1, and centrifuged for 15 minutes in a centrifuge with a horizontal rotor at a speed of 1500 rpm. Then an interphase ring containing lymphocytes was collected and washed with 7–10 ml of saline. Next, the cells were pelleted by centrifugation for 10 min at 1500 rpm, resuspended in saline and the number of cells in the resulting suspension was determined using a Goryaev chamber by staining them with 0.4% trypan blue solution. The isolated cells were seeded in the wells of a 6-well plate in RPMI 1640 medium with 10% ECT and 40 μg/ml gentamicin at a concentration of 1.5 × 10^6^ cells/ml, 3 ml per well (4.5 × 10^6^ cells/well). Inserts for culturing with a pore size of 0.4 nm were placed in all wells of the plates at once and 2 ml of RPMI 1640 medium with 10% ECS and 40 μg/ml gentamicin were added to the inserts. Next, “Activegel” was added to the corresponding inserts in a quantity of 0.2 g per 1 ml of nutrient medium. The cells with the test substance were incubated in a CO_2_ incubator for another 96 hours. After incubation, the cells were washed twice with saline by centrifugation at 1500 rpm for 10 minutes. The resulting cell suspension was immediately used in the DNA comets assay and micronuclei test (MN test).

##### Gel electrophoresis of single cells (DNA comet assay)

The DNA comet assay was performed in an alkaline modification developed by Singh and colleagues [18]. For positive control cells were treated with 50 μM H_2_O_2_ for 5 min, for negative control – with saline solution in the same amount as “Activegel”, as described above. The first layer of gel contained a 1% solution of high melting agarose, pre-treated overnight in a thermostat at 37 °C. 200 μl of the cell suspension (5 × 10^5^ cells/ml) were mixed with 200 μl of 1% low melting agarose at 37 °C. This suspension was applied on the preheated glass slide coated with high-melting agarose and kept at + 4 °C until the gel was completely solidified. Cell lysis was performed in lysis buffer (2.5 M NaCl, 0.1 M EDTA, 10 mM Tris pH 10, 10% DMSO, 1% Triton X-100) at +4^0^С for 1 hour. Before electrophoresis, the slides were washed with cold water and kept for 20 minutes in electrophoretic buffer (0.3 M NaOH, 1 mM Na_2_EDTA). Electrophoresis was performed at ≈ 0.8 V/cm (distance between electrodes 27 cm, voltage 24 V, current 300 mA for 30 minutes). Neutralization was performed in appropriate buffer (0.4 M Trizma base, pH 7.5) for 10 minutes.

##### Analysis of DNA comets

After drying, the samples were stained with acridine orange (2 μg/ml) and SYBR, 50–100 μl per sample. Microscopic analysis of micropreparations was performed using a fluorescence microscope (Axiostar Plus Microscope (Carl Zeiss, Germany), Digital Camera (Canon powershot G5, UK), fluorescent lamp (Carl Zeiss, Germany)). At least 50 DNA comets per micropreparation were analyzed. Digital images were analyzed using the computer program CometScore. The parameters of the length of the comet’s tail, the moment of the tail, as well as the proportion of DNA in the comet’s tail were calculated.

##### Micronuclei test

Slides were prepared according to standard methods: before and 48 hours after exposure to “Activegel” and saline solution (control) human peripheral blood lymphocytes were kept in hypotonic solution (0.9% sodium citrate solution) for 40 minutes at 37 °C. Fixation was performed with a mixture of methyl alcohol and acetic acid in a ratio of 3: 1, changing the retainer three times. The cell suspension was applied to slides and after drying was stained with acridine orange (2 μg/ml) dye.

##### Slides analysis

The frequency of cells with micronuclei were calculated in the obtained cytological slides. 1000 cells for each sample were analyzed in three repeats, the results were expressed in MN number per mile (‰). Microscopic analysis was performed using a fluorescence microscope (Axiostar Plus Microscope (Carl Zeiss, Germany), Digital Camera (Canon powershot G5, UK), fluorescent lamp (Carl Zeiss, Germany).

### Statistical analysis

Calculations of the mean values ​​of the studied indices (M), standard deviation (SD) and standard error of the mean (m) were performed using Excel 2016 software package and Medstatistic program using Student’s t-test; differences with a probability of at least 95% (*p* < 0.05) were considered significant. The nonparametric Mann-Whitney U-test was used to assess the significance levels of the differences between the mean values ​​between the groups. The calculations were performed using the software package STATISTICA 6.0. Differences were considered significant at *p* < 0.05.

## Results and discussion

The first step was to assess the acute toxicity of the medical product “Activegel” in vivo to understand and confirm the data on the test substance (stated by the manufacturer) on the absence of its toxicity. During the experiment, the survival and physiological parameters of rats (males and females) in the control and experimental groups were analyzed. During 48 hours (stage I) and 14 days (stage II) we have analyzed locomotor and respiratory activity of experimental animals, the condition of fur, mucous membranes, behavior, usage of food and water, and gastrointestinal function.

We have found that the highest of the studied doses of “Activegel” (5000 mg/kg) did not cause the death of animals. In the control and all experimental groups of male rats within 48 and 14 days after the 1st injection of the test substance, no deviations in the studied indices of the physiological state were observed. However, immediately after injection of saline or test substance and for the next 15 minutes in 50–100% of the animals the signs of aggression were observed. Since aggressive behavior of animals was observed in both control and experimental groups, it is clear that it was not a reaction to the test substance, but to the procedure of forced fixation of rats for injection procedure. In groups of female rats, which were administered different doses of “Activegel” or saline, no violations of the physiological state of the experimental animals were observed during the entire observation period.

Analysis of the dynamics of body weight changes in rats within 48 hours and 14 days after single subcutaneous injection of “Activegel” at different doses did not reveal statistically significant differences in the studied index compared with the control group: animal body weight in the experimental groups increased as well as in the control.

Macroscopic examination of the condition of the internal organs of experimental animals and the condition of the tissues at the injection site after single subcutaneous administration of “Activegel” at different doses showed that it did not affect the internal organs of the animals.

**Fig. 1:**
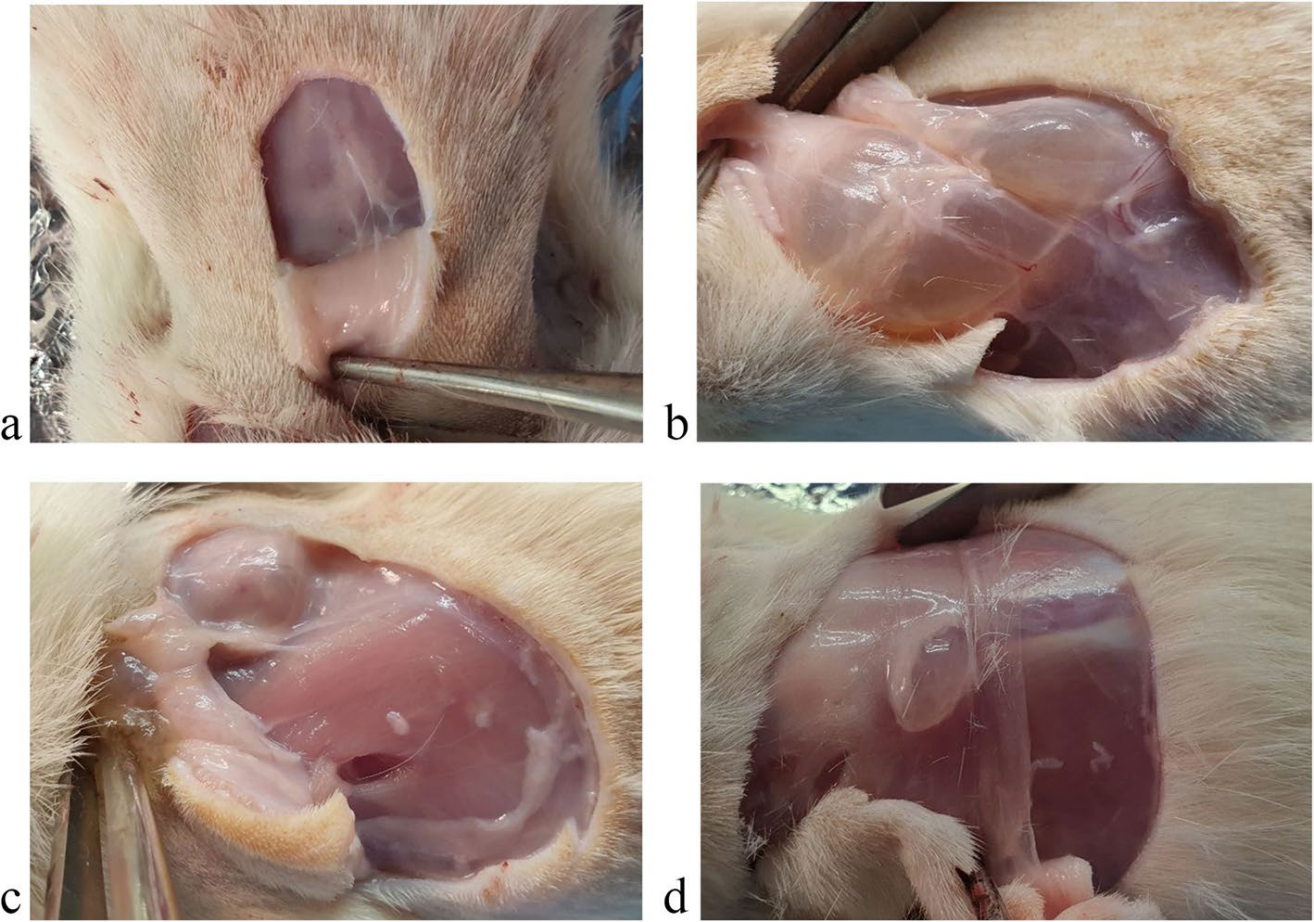
Photo of the site of localization of the substance “Activegel” in the skin tissues of male rats 14 days after injection of the product. **A** – control group, **B** - + “Activegel” 5000 mg/kg, **C** - + “Activegel” 2000 mg/kg, **D** - + “Activegel” 500 mg/kg

**Fig. 2:**
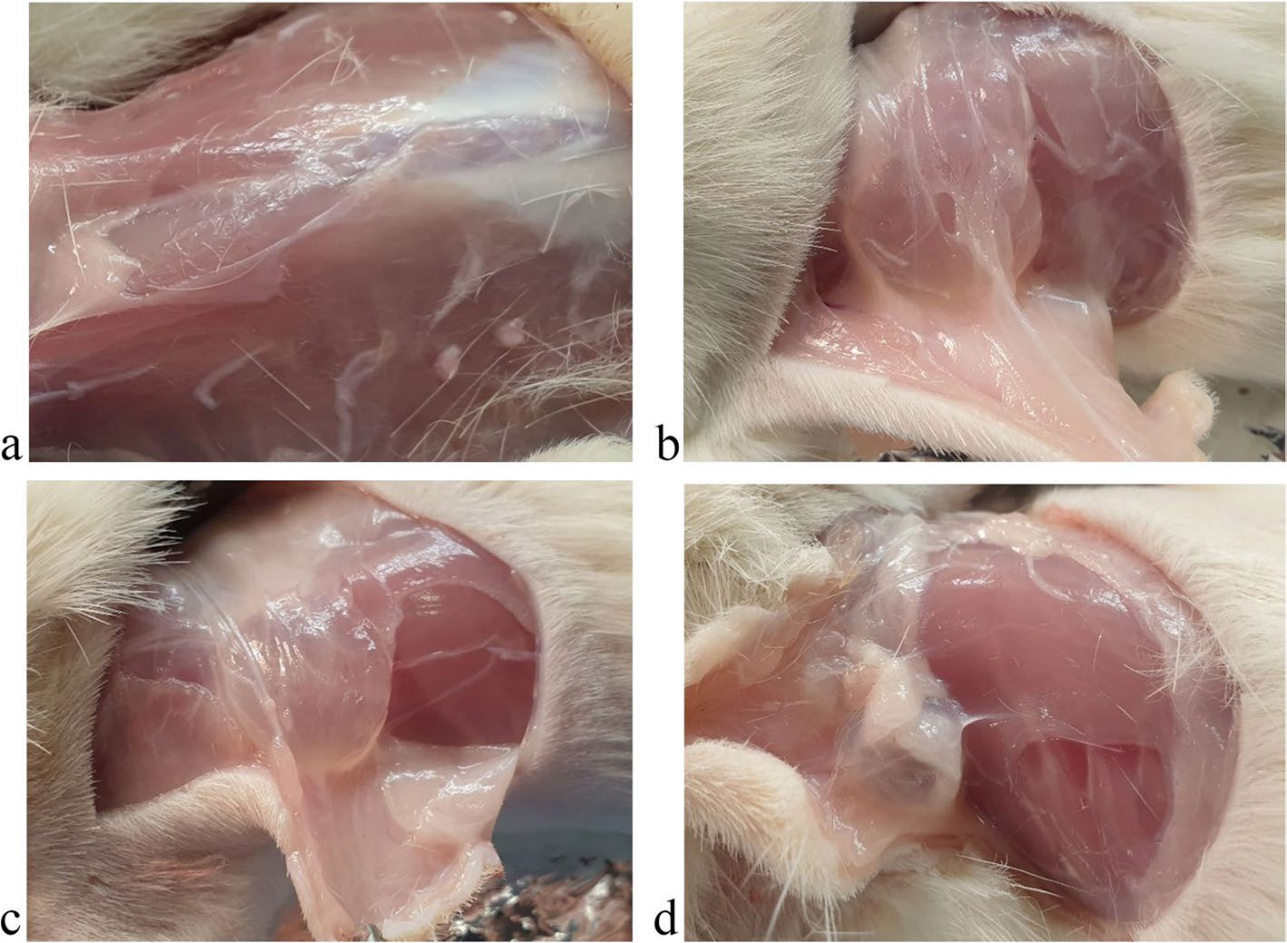
Photo of the site of localization of the substance “Activegel” in the skin tissues of female rats 14 days after injection of the product. **A** – control group, **B** - + “Activegel” 5000 mg/kg, **C** - + “Activegel” 2000 mg/kg, **D** - + “Activegel” 500 mg/kg

One of the most important stages of the study of medicinal products is the macroscopic analysis of the condition of the tissues located in the injection site of the test substance, because the characteristics of such contact areas are indicative of its aggressiveness. We showed the absence of signs of inflammation and abnormalities in the condition of the studied tissues in experimental animals (Figs. 1 and 2).

**Fig. 3:**
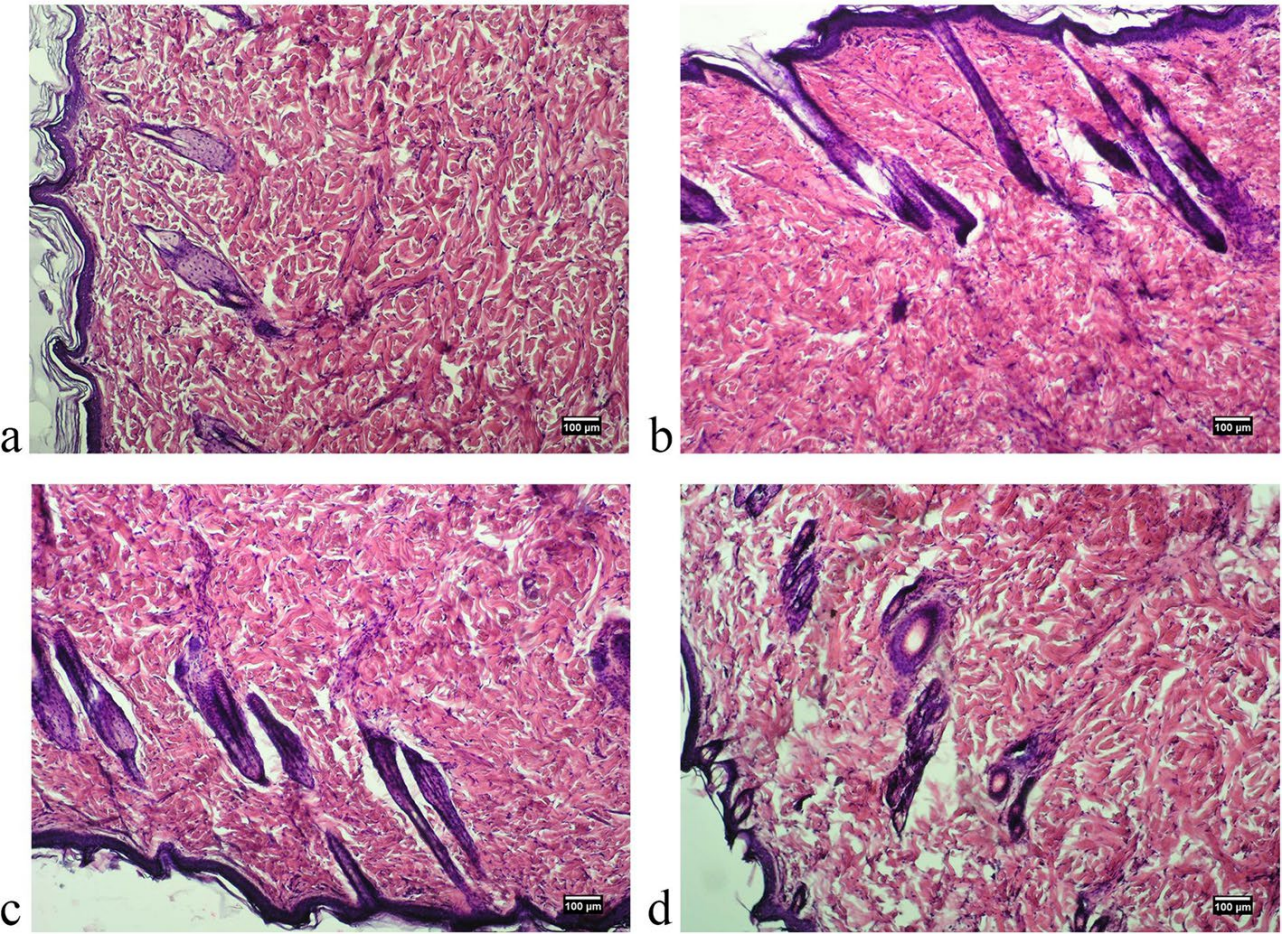
Microphotographs of skin of a male rat 48 hours after treatment: **A**. control group, **B**. “Activegel” at a dose of 500 mg/kg., 2000 mg/kg (**C**) and 5000 mg/kg (**D**). Hematoxylin and eosin staining (× 100)

**Fig. 4:**
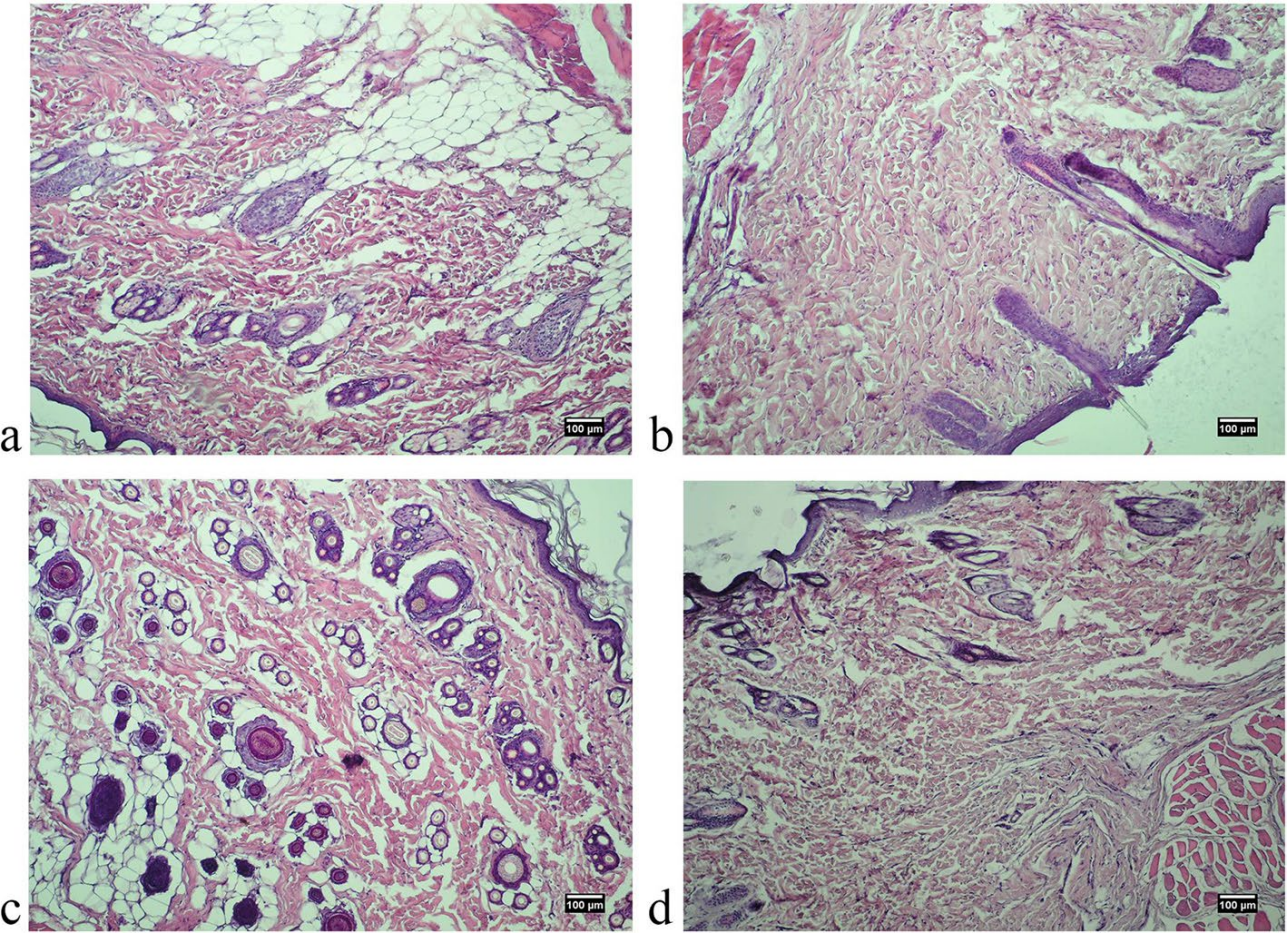
Photographs of skin of a female rat 48 hours after treatment: **A**. control group, **B**. “Activegel” at a dose of 500 mg/kg., 2000 mg/kg (**C**) and 5000 mg/kg (**D**). Hematoxylin and eosin staining (× 100)

**Fig. 5:**
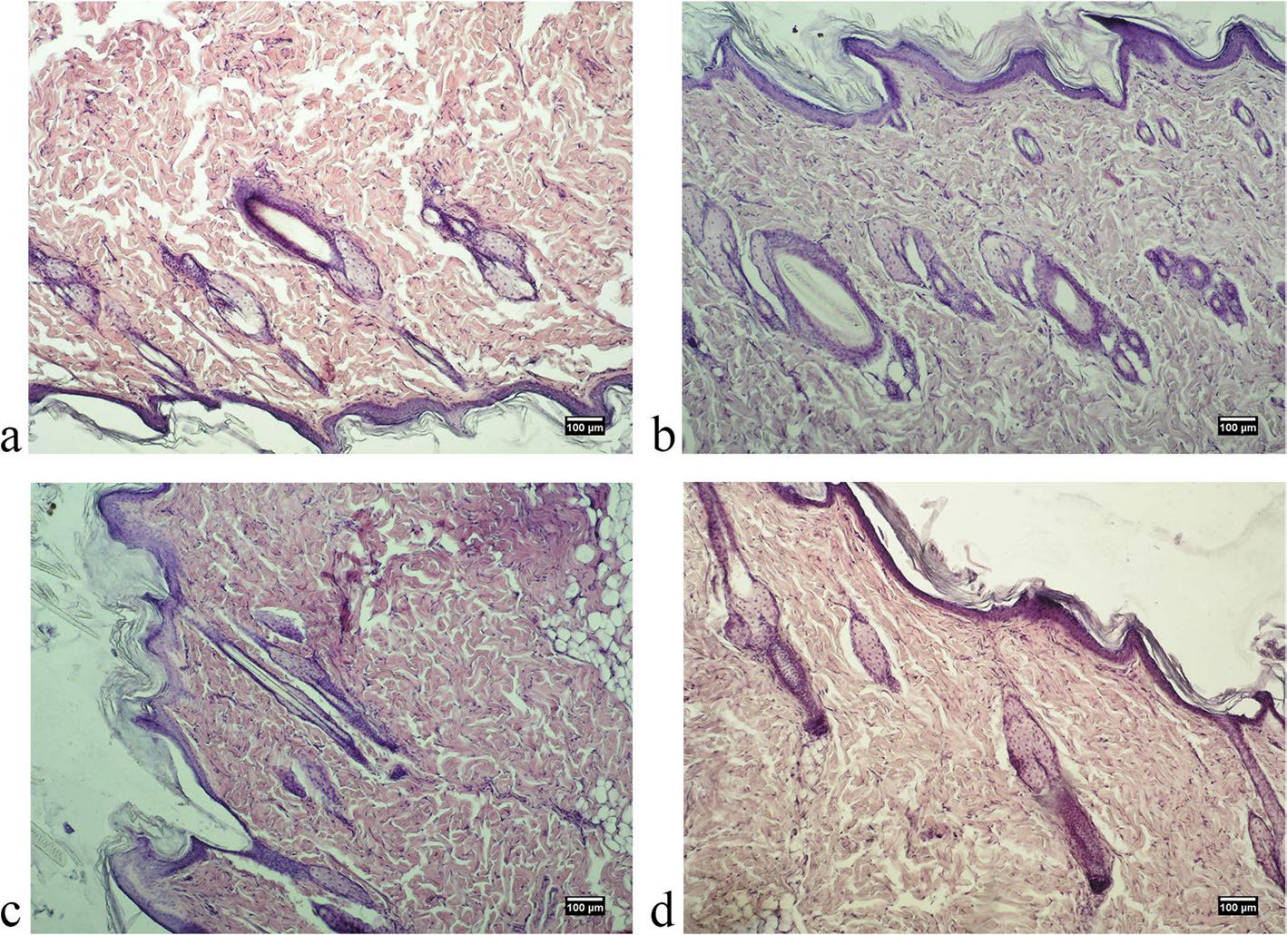
Microphotographs of skin of a male rat 14 days after treatment: **A**. control group, **B**. “Activegel” at a dose of 500 mg/kg., 2000 mg/kg (**C**) and 5000 mg/kg (**D**). Hematoxylin and eosin staining (× 100)

**Fig. 6:**
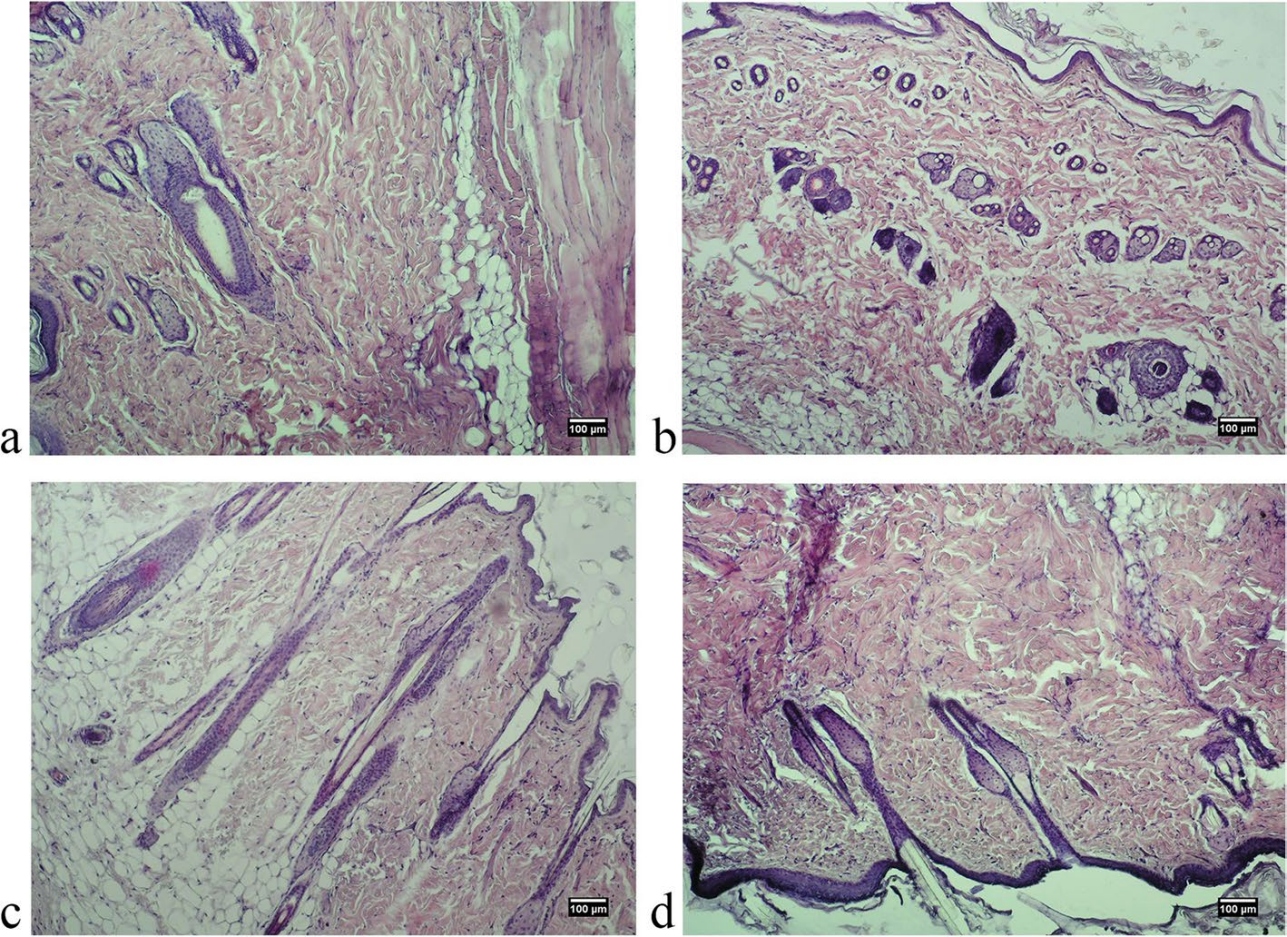
Microphotographs of skin of a female rat 14 days after treatment: **A**. control group, **B**. “Activegel” at a dose of 500 mg/kg., 2000 mg/kg (**C**) and 5000 mg/kg (**D**). Hematoxylin and eosin staining (× 100)

Pathomorphological examination of skin samples of all experimental animals of both sexes showed no pathological changes: no redness, hemorrhage, rupture, pus, edema, the presence of foreign bodies etc. (Figs. 3, 4, 5 and 6). Morphological examination showed normal histoarchitectonics of the skin in all groups of animals of both sexes. In the skin there was a clear division into the epidermis and the dermis. Under the skin, subcutaneous fat and a layer of the skin muscle fibers were visible. The epidermis was represented by a multilayered keratinized epithelium lying on a distinct basement membrane that was uniform in thickness, without ruptures or thinnings. Maturity and zonation of the basal, prickle and granular layers of the epidermis without signs of inflammatory infiltrate were clearly traced. The papillae of the papillary layer of the dermis of all experimental groups were clearly expressed and deeply immersed in the epithelial layer. In the papillary layer there was loose connective tissue with vessels without pathological signs. No signs of inflammation in the papillary layer were found in any of the experimental groups.

The reticular layer of the dermis is represented by a dense unformed connective tissue. Fibroblasts were present in significant quantities in the dermis of all test specimens; the number of fibroblasts and collagen fibers indicates active collagenosis. There were no signs of edema in the reticular layer. By the nature of collagen fibers, no signs of scarring or fibrosis were detected, and the blood supply to the skin of all experimental animals was normal. The condition of the vessels of the dermis was normal, without hemostasis, signs of dystonia or spasms: no vascular hyperemia, vascular endothelium is not changed, not thickened. No inflammatory lymphocyte infiltration (neither diffuse nor local infiltration) was detected in the papillary and reticular layers of the dermis in any sample at all observation time points. In addition, the absence of eosinophils and macrophages in the dermis indicated the absence of an allergic reaction to the implant.

In the reticular layer of the dermis of all experimental animals a large number of mature hair follicles were preserved. The follicles were not deformed, without signs of keratinocyte hyperplasia. Next to the follicles there were often visible blood vessels, the endothelium of which exerted no pathological changes. Also, all groups of animals had a large number of sebaceous glands, the structure of which corresponded to the physiological norm. The hypodermis was formed by clusters of adipocytes without signs of edema or redness.

The obtained results indicated a normal morpho-functional state of the skin tissues of experimental animals of both sexes at the location of the implanted substance irrespectively of the doses and timing of implantation.

Thus, according to the study of in vivo toxicity of “Activegel”, no clinical manifestations of intoxication were observed in animals of all experimental groups: no deviations in the studied indices of the physiological state of rats, changes in the weight of animals and their internal organs, or disorders of their internal organs. The normal condition of animal skin tissues at the injection site and the absence of systemic and local markers of inflammation were registered.

The results of general and biochemical blood tests of experimental animals of both sexes 48 hours after injection of the test substance indicated the absence of significant changes in most of the studied indices. However, in the group of male rats injected with “Activegel” at a dose of 2000 mg/kg, biochemical analysis of peripheral blood revealed a significantly lower glucose concentration compared to the control. In the group of male rats treated with “Activegel” at a dose of 500 mg/kg, there was registered a significantly lower ALT activity (by 16.2%) compared with the control (*р* < 0.05). It is important to note that the above changes in indices, although different from the control group, were within normal range (https://en.wikivet.net/Rat_Haematology) [19–21].

Assessment of the effect of “Activegel” on the indices of general and biochemical blood tests of female rats revealed a significant increase in the leukocyte counts (by 26.4%, compared with the control, *p* < 0.05) only in the group treated with the highest dose (5000 mg/kg). It should be noted that in this group of animals the increase in the leukocyte count was not accompanied by changes in the leukocyte formula. Therefore, the results suggest that this is a physiological leukocytosis, a temporary phenomenon not associated with disorders of the body.

In all groups of female rats administered with “Activegel”, an increase in the concentration of inorganic phosphorus was observed by 31% (5000 mg/kg) (*р* < 0.05), 25% (2000 mg/kg) (*р* < 0.05) and 37.5% (500 mg/kg) (*р* < 0.05) compared with the control group. Phosphorus increase was observed only in females, while in males there were no changes and in comparison, the highest level of phosphorus in females was even lower than in males and no changes in calcium were observed (which are interrelated indicators). In our opinion, such an increase could be related to certain nutritional or hormonal temporal features of female rats.

In the group of female rats injected with the test substance at a dose of 500 mg/kg, a statistically significant decrease in the concentration of urea in the blood by 15% (*р* < 0.05) was registered, compared with the control group.

The results of general and biochemical blood tests of animals of both sexes 14 days after single injection of the test substance indicated the absence of significant changes in most of the studied indices. However, in the group of male rats injected with the test substance at a dose of 5000 mg/kg, a significant (*р* < 0.05) increase in glucose concentration by 26.3% and ionized calcium by 9% was observed, compared with the control group. In the group of male rats injected with “Activegel” at a dose of 2000 mg/kg, general and biochemical analysis of peripheral blood revealed the significantly higher concentrations of hemoglobin (by 6.8%), glucose (by 26.3%) and ionized calcium (by 9%) compared to the control. In the group of male rats administered with “Activegel” at a dose of 500 mg/kg we registered a significantly lower hemoglobin concentration (4.8%) and higher glucose concentration (31.6%) compared with the control. One should note that the above changes in the indices of general and biochemical blood analysis of of rats, although different from the control group, remained within normal range [19, 20, 22, 23].

It should be noted that in the groups of male rats administered with the test substance at doses of 2000 mg/kg and 500 mg/kg, there was also observed a significant increase in the concentration of total bilirubin by 35.7% (*р* < 0.05) and 42.9% (*р* < 0.05), respectively, compared with the control. As far as in animals of the these groups moderate but significant differences in hemoglobin concentration were registered, it’s reasonable to suppose that the increase in total bilirubin concentration could be associated with physiological processes of hemoglobin synthesis/breakdown in experimental animals.

According to the results of the general blood analysis of female rats, a significant increase in the leukocyte counts by 34.5% and two-fold increase of monocyte counts was found compared with the control, in animals treated only with the largest of the studied doses (5000 mg/kg) of the test substance. In this group of animals, leukocytosis was accompanied by changes in the leukocyte formula, in particular, a small increase in the % content of monocytes, which may indicate the reactivity of the organism in response to the introduction of a large dose of test substance.

In the group of female rats injected with Activegel at a dose of 2000 mg/kg, biochemical analysis of peripheral blood revealed only a significantly higher concentration of ionized calcium by 9%, compared with the control group. In the group of female rats injected with the test substance at a dose of 500 mg/kg a significantly lower glucose concentration and higher concentration of inorganic phosphorus (46.7%) was observed compared with the control. It is important to note that these altered biochemical parameters, according to the literature, were within normal range [20, 23].

Thus, the in vivo toxicity of the new generation hydrophilic gel was evaluated using generally accepted approaches to clinical trials of the compounds and it was shown that this substance, according to the classification of the Environmental Protection Agency can be classified as hazard category IV, is not toxic. The data obtained by us can be used for further preclinical and clinical studies on the medical use of new generation hydrophilic gel “Activegel”.

As the lack of toxicity of the test substance in vivo was shown, to expand the understanding of its safety, certain in vitro studies of “Activegel” have been planned. The next step was to study its possible genotoxic effect in vitro using the analysis of human peripheral blood lymphocytes by DNA comet assay after their exposure to the test substance.

**Fig. 7:**
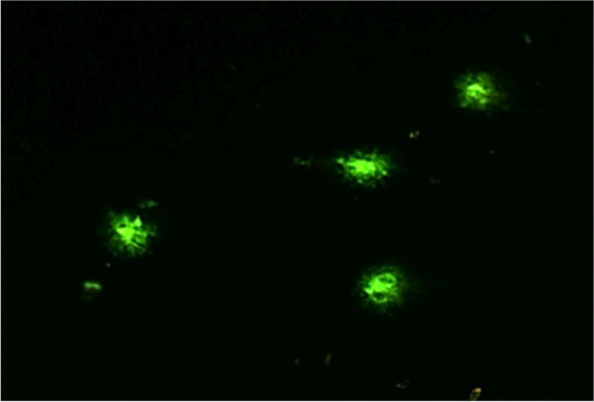
A typical field of view with several DNA comets in the analysis, 400x

**Fig. 8:**
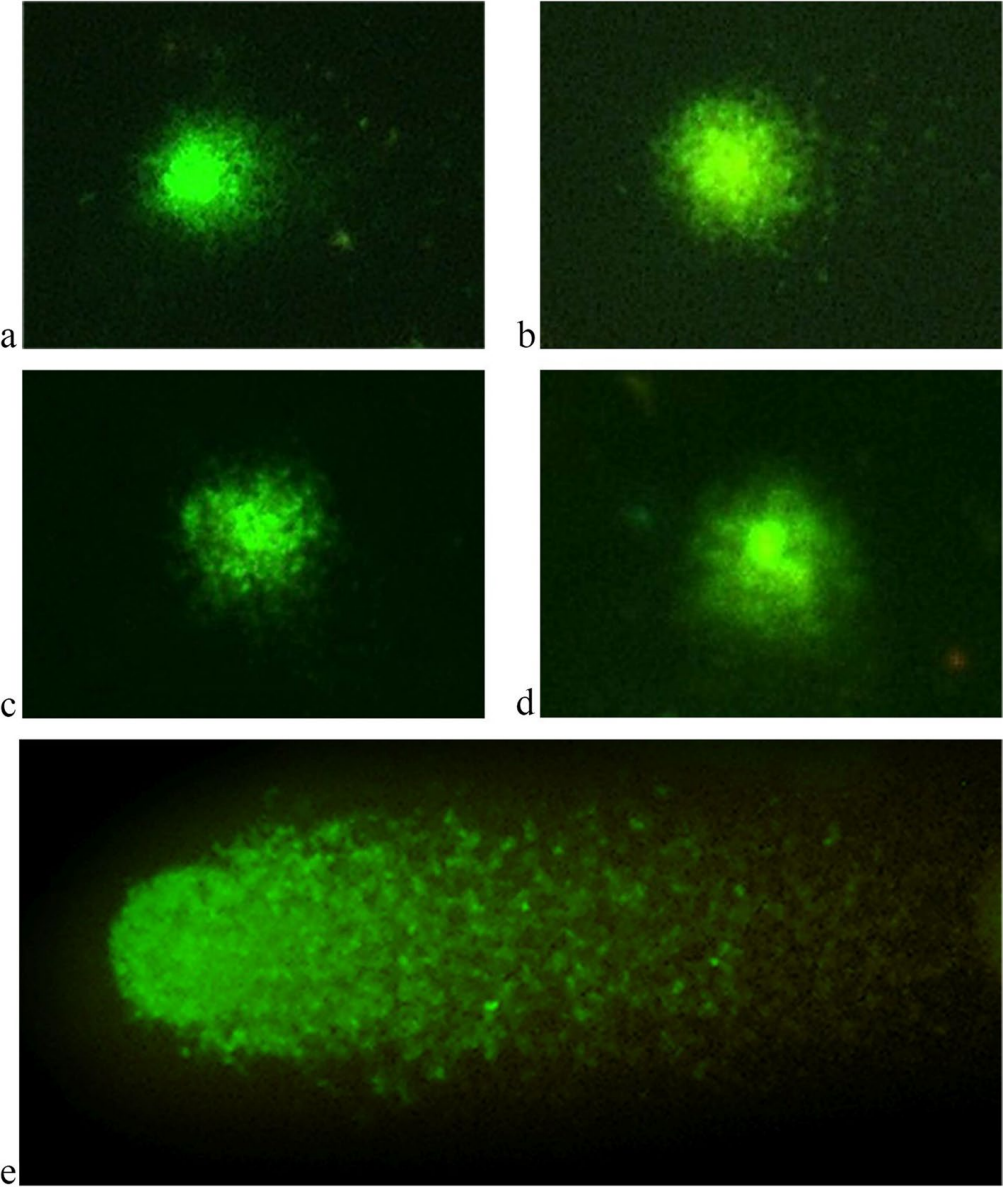
The nuclei of cells with a low % yield of DNA in the comet tail. **A**, **B**. - Control (human peripheral blood lymphocytes + saline); **C**, **D** - human peripheral blood lymphocytes + “Activegel”; **E** – positive control human peripheral blood lymphocytes + H_2_O_2_

Analysis of the DNA integrity in peripheral blood lymphocytes of healthy donors cultured in the presence of “Activegel” at a concentration of 0.2 g of the substance per 1 ml of nutrient medium showed that the test substance exerted no genotoxic effects on the cells. The studied parameters of DNA comets in the experimental group (the amount of DNA in the “tail” of comets, the length and moment of the tail) did not differ from the parameters of the control (cells treated with saline) Comet parameters in positive control study also did not differ between donor samples (Table 1, Figs. 7 and 8).

**Table 1:** Analysis of the parameters of DNA comets of human peripheral blood lymphocytes after their cultivation in the presence of a medical product “Activegel” in vitro

Experimental group	Parameters of DNA comets		
	Tail length, px	% DNA in tail	Tail moment
**Peripheral blood lymphocytes + saline, Control**			
Donor №1	85.71 ± 5.6	0.25 ± 0.02	0.27 ± 0.03
Donor №2	97.31 ± 2.0	0.20 ± 0.01	0.25 ± 0.02
Donor №3	82.07 ± 3.5	0.17 ± 0.01	0.20 ± 0.02
Donor №4	83.52 ± 6.6	0.20 ± 0.05	0.21 ± 0.01
М ± m	**87.2 ± 6.9**	**0.21 ± 0.03**	**0.23 ± 0.03**
**Peripheral blood lymphocytes + “Activegel”**			
Donor №1	93.96 ± 3.0	0.22 ± 0.02	0.22 ± 0.01
Donor №2	72.96 ± 8.5	0.15 ± 0.02	0.23 ± 0.0
Donor №3	96.97 ± 0.4	0.19 ± 0.02	0.22 ± 0.09
Donor №4	104.14 ± 17.1	0.27 ± 0.12	0.21 ± 0.04
М ± m	**92.0 ± 13.4**	**0.21 ± 0.05**	**0.22 ± 0.01**
**Peripheral blood lymphocytes + H**_**2**_**O**_**2**_**, Positive control**			
Donor №1	258.46 ± 15.38	44.46 ± 2.79	70.20 ± 8.79
Donor №2	269.23 ± 17.59	53.04 ± 5.64	82.76 ± 5.47
Donor №3	292.31 ± 20.68	52.26 ± 5.49	77.21 ± 6.79
Donor №4	223.77 ± 26.49	45.24 ± 3.77	69.26 ± 6.49
М ± m	**260.76 ± 24.96**	**48.75 ± 3.92**	**74.85 ± 5.50**

In the experiment, a small “tail” of the comet was observed, which evidenced on of the presence of a low number of single-stranded DNA breaks. However, the low level of luminescence and the large area of the “tail” of the comet are characteristic of cases where nucleic acid breaks can be attributed to “junk DNA”, which is a variant of the norm [24]. Therefore, these results indicated that “Activegel” in the studied concentration (0.2 g/ml) did not affect the parameters of DNA comets, ie the test substance exerted no genotoxic effect on the cells.

**Fig. 9:**
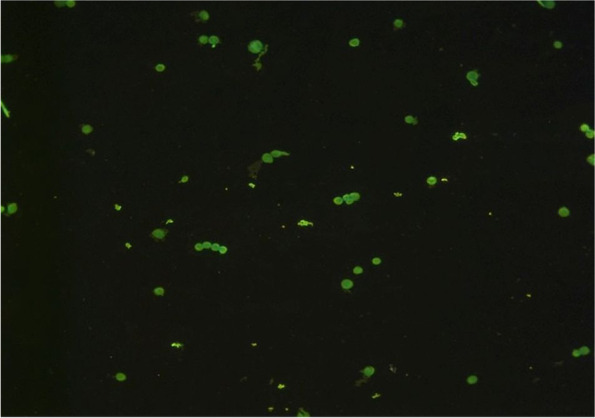
Typical field of view with human peripheral blood lymphocytes, 40x

**Fig. 10:**
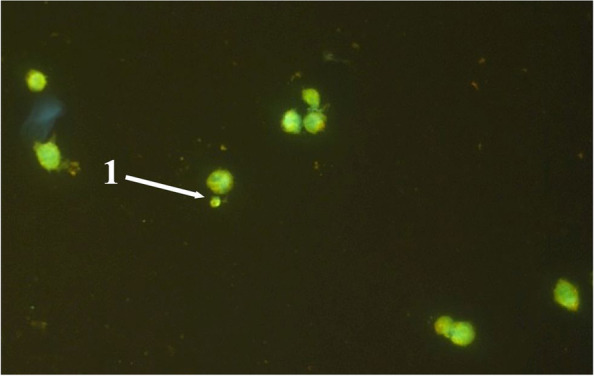
Typical field of view with human peripheral blood lymphocytes, 1-cell with micronuclei, 100x

Analysis of human peripheral blood lymphocyte slides after cultivation with “Activegel” and saline solution (control) proved that the drug does no effect the formation of micronuclei in examined cells, and therefore has no genotoxic effect (Table 2, Figs. 9 and 10).

**Table 2:** Results of MN test of the human peripheral blood lymphocytes after their cultivation in the presence of a medical product “Activegel” in vitro

Experimental group	MN (‰)
**Peripheral blood lymphocytes + saline, Control**	
Donor №1	6.0 ± 1.0
Donor №2	5,6 ± 1,5
Donor №3	7,1 ± 1,3
Donor №4	6,5 ± 1,5
**М ± m**	**6.3 ± 0.65**
**Peripheral blood lymphocytes + “Activegel”**	
Donor №1	6,2 ± 0,7
Donor №2	5,3 ± 0,9
Donor №3	6.8 ± 0,6
Donor №4	5,9 ± 0,6
**М ± m**	**6.0 ± 0.62**

The obtained data on the absence of genotoxicity on human lymphocytes are an additional positive characteristic of the new generation hydrophilic gel for further expanding of its use. Because this substance can potentially be used as an implant in breast reconstruction in breast cancer patients, we conducted the study on the effect of “Activegel” on breast cancer cells of varying degrees of malignancy, MCF-7 and MDA-MB-231 cell lines. Moreover, this hydrophilic gel (as well as its known analogues) can be potentially used as a “vector” for targeted delivery of biologically active molecules, including anticancer drugs, therefore an assessment of its effect on tumor cells is really important. In this work, we have studied the effect of breast cancer cells exposure to the gel on their proliferative activity (Tables 3 and 4).

**Table 3:** The number of living cells of MDA-MB-231 line after the action of “Activegel”

% of conditioned nutrient medium	MDA-MB-231 + saline solution			MDA-MB-231 + “Activegel”		
	№1	№2	№3	№1	№2	№3
	Number of living cells, %					
	48 h					
100	100.4 ± 1.8	102.7 ± 3.2	103.6 ± 3.5	104.3 ± 3.9	102.2 ± 4.3	99.7 ± 3.8
50	104.0 ± 4.4	106.1 ± 0.2	98.0 ± 0.9	100.4 ± 3.7	101.8 ± 4.7	101.9 ± 2.4
25	101.6 ± 3.5	103.3 ± 3.1	102.0 ± 3.1	103.1 ± 2.9	103.3 ± 2.8	100.5 ± 2.1
12.5	100.7 ± 0.9	100.8 ± 5.0	99.6 ± 4.4	101.5 ± 3.8	103.9 ± 4.1	100.8 ± 3.1
6.3	101.9 ± 1.0	102.5 ± 3.5	101.0 ± 2.8	101.0 ± 3.8	103.4 ± 1.9	99.6 ± 1.8
3.1	103.2 ± 2.6	102.1 ± 2.8	101.7 ± 3.4	100.6 ± 2.3	103.2 ± 1.8	102.8 ± 2.6
1.6	104.4 ± 2.2	103.3 ± 2.3	99.3 ± 1.8	99.6 ± 0.7	100.9 ± 3.1	100.5 ± 1.6
	96 h					
100	100.8 ± 2.3	100.0 ± 3.0	101.7 ± 3.7	98.3 ± 1.1	100.4 ± 3.6	99.1 ± 3.2
50	99.0 ± 4.1	99.7 ± 1.6	100.1 ± 5.0	100.9 ± 4.1	102.1 ± 3.1	101.4 ± 2.7
25	100.9 ± 3.6	98.7 ± 3.0	104.6 ± 1.5	102.7 ± 2.5	103.1 ± 2.0	101.1 ± 5.0
12.5	101.7 ± 3.2	100.6 ± 0.9	103.7 ± 2.6	102.5 ± 3.0	103.1 ± 1.5	102.7 ± 2.4
6.3	99.5 ± 1.8	100.9 ± 1.9	101.5 ± 3.6	102.7 ± 3.8	101.4 ± 2.9	101.0 ± 2.2
3.1	102.8 ± 2.9	100.6 ± 4.0	102.5 ± 1.9	102.4 ± 3.6	99.0 ± 2.9	102.3 ± 2.6
1.6	98.3 ± 2.5	102.9 ± 2.2	99.9 ± 2.6	101.1 ± 2.1	99.6 ± 1.2	98.7 ± 3.1

**Table 4:** The number of living cells of MCF-7 line after the action of “Activegel”

% of conditioned nutrient medium	MCF-7 + saline solution			MCF-7 + “Activegel”		
	№1	№2	№3	№1	№2	№3
	Number of living cells, %					
	48 h					
100	101.3 ± 2.3	97.2 ± 1.5	100.3 ± 0.5	101.5 ± 2.4	101.1 ± 3.0	99.0 ± 1.7
50	99.9 ± 0.8	99.9 ± 2.3	98.7 ± 1.6	100.0 ± 0.9	100.6 ± 2.7	105.0 ± 1.8
25	99.9 ± 2.9	100.3 ± 3.2	102.3 ± 2.7	98.1 ± 1.1	101.2 ± 1.4	100.2 ± 0.7
12.5	100.8 ± 1.9	99.6 ± 0.5	99.2 ± 1.8	100.3 ± 3.2	99.3 ± 3.6	101.0 ± 1.8
6.3	101.7 ± 3.2	101.3 ± 2.9	100.0 ± 1.9	99.7 ± 2.0	101.8 ± 4.2	102.6 ± 2.2
3.1	99.3 ± 2.5	102.2 ± 3.1	99.9 ± 1.5	101.6 ± 1.4	99.2 ± 1.5	101.4 ± 2.7
1.6	99.3 ± 2.8	101.6 ± 1.1	98.7 ± 1.4	100.1 ± 2.2	101.9 ± 2.3	98.7 ± 3.7
	96 h					
100	100.3 ± 2.0	99.6 ± 2.1	100.3 ± 1.8	99.8 ± 3.1	99.1 ± 1.0	99.4 ± 3.1
50	102.2 ± 2.1	99.0 ± 3.4	99.7 ± 0.5	101.0 ± 3.1	99.2 ± 1.7	100.4 ± 2.7
25	99.5 ± 2.1	101.0 ± 1.3	103.5 ± 1.3	100.0 ± 1.0	101.3 ± 1.3	100.1 ± 0.4
12.5	101.0 ± 2.2	102.0 ± 2.1	100.8 ± 2.6	102.8 ± 1.4	98.4 ± 1.2	101.9 ± 2.8
6.3	100.5 ± 2.0	102.0 ± 2.2	101.2 ± 1.9	99.4 ± 2.9	102.3 ± 3.3	101.7 ± 2.9
3.1	101.3 ± 2.9	103.2 ± 1.6	100.9 ± 2.8	99.7 ± 1.5	100.0 ± 0.2	103.2 ± 2.3
1.6	98.3 ± 3.7	104.0 ± 3.0	103.4 ± 2.2	98.0 ± 2.2	99.2 ± 1.2	99.0 ± 2.5

According to the study, it was determined that the new generation hydrophilic gel for implantation does not affect either the proliferative activity or the viability of cells (Tables 3 and 4) of human breast cancer in the in vitro system, which potentially indicates its inertness (but, in our opinion, requires further more detailed clinical trials when used as an implant after surgery for patients with a history of breast cancer).

There was no difference between the effect of the test gel on malignantly transformed cells of varying degrees of malignancy: the inertness of the action of “Activegel” was observed when assessing the growth characteristics of both cell lines (MCF7, and MDA-MB-231). The findings are highly important because they indicate the potential safety of this product in cancer patients, but such statements require further more detailed study of the effect of “Activegel” on tumor cells (including their phenotypic characteristics and tumorigenicity). Such research is important, because the revealed lack of toxicity of the hydrophilic gel and the inertness of its action on tumor cells opens up new possibilities for the use of this product as a vector for targeted therapy.

In our opinion, this direction of application of hydrophilic gel (“Activegel”) looks quite promising, as the known ability of hydrogel to encapsulate and release small molecules and biologicals is currently being actively implemented for synthetic preparations (used as a “depot system with targeted delivery”). In particular, there are known such developments as “Endo’s Vantas ®” approved by the FDA for subcutaneous hormone therapy to prevent the growth of hormone-dependent prostate cancer cells, and “SpaceOAR®” hydrogel (Augmenix) for the protection of prostate cancer patients undergoing radiation therapy [14].

However, to date, none of the natural hydrogels (as opposed to synthetic) have been approved as targeted delivery systems for antitumor drugs, which may be due to the advantages of synthetic hydrogels due to the possibility of prolonged release of bioactive molecules, i.e. significant prolongation of local action. The benefits of using synthetic hydrogels are also evidenced by the data from “TraceIT®” and “SpaceOAR®”, which are widely studied to visualize tumor cells and protect normal cells from radiation damage [14].

## Conclusion

Under the conditions of subcutaneous implantation to rats, the gel “Activegel” did not cause the death of experimental animals even at the highest of the studied doses (5000 mg/kg). In animals from all experimental groups there were found no deviations of the studied indices of the physiological state, weight, state of internal organs, general and biochemical blood tests, compared with the control group. The morpho-functional condition of the skin tissues of experimental animals of both sexes at the site of the gel implantation at different doses and different time points remained normal, and systemic and local markers of inflammation were absent. The results of the genotoxicity test indicated that the studied gel for implantation did not affect the parameters of DNA comets and the formation of micronuclei, accordingly, exerted no genotoxic effect on human lymphocytes. In the in vitro study it was demonstrated that “Activegel” did not affect the proliferative activity and viability of human breast cancer cells, i.e. is characterized by inertness to tumor cells and can be recommended for further preclinical/clinical studies as implant material and a potential vector for targeted cancer therapy.

## Supplementary Information

**Additional file 1.**

## Data Availability

The datasets used and/or analysed during the current study are available from the corresponding author on reasonable request.

## Abbreviations

ALT: Alanineaminotransferase
AST: Aspartate aminotransferase
DMSO: Dimethyl sulfoxide
*DNA*: Deoxyribonucleic acid
EDTA: Ethylenediaminetetraacetic acid
ESR: Erythrocyte sedimentation rate
FCS: Fetal calf serum
IEPOR: Institute of Experimental Pathology, Oncology and Radiobiology
LLC: Limited liability company
MN: Micronuclei
NAS: National Academy of Sciences
