# Supplementary material for: Assessment of biosafety and toxicity of hydrophilic gel for implantation in experimental in vitro and in vivo models
Source: BMC Pharmacol Toxicol. 2022 Jun 8;23:37. doi: 10.1186/s40360-022-00577-3 (PMC9178808; doi:10.1186/s40360-022-00577-3)
Supplement: Supplementary file 1 — Additional file 1. [file 40360_2022_577_MOESM1_ESM.docx]

Table 1

**Evaluation of the effect of "Activegel" on the biochemical parameters of the peripheral blood of male rats 48 hours after 1 subcutaneous injection**

| Group number | Biochemical parameters of peripheral blood of experimental animals | | | | | | | | | | |
| --- | --- | --- | --- | --- | --- | --- | --- | --- | --- | --- | --- |
|  | АLТ, u/l | АSТ, u/l | Total bilirubin, μmol / l | Calcium, mlmol / l | Creatinine,  μmol / l | Glucose, mlmol / l | Inorganic phosphorus, mmol / l | Total protein, g / l | Urea, mlmol / l | C-reactive protein, mlg / l | Ca +++, mlmol / l |
| 1  control | 74,3±4,2 | 159,5±20,0 | 1,6±0,4 | 2,5±0,1 | 37,8±1,8 | 9,8±0,4 | 2,3±0,2 | 63,2±2,4 | 9,0±0,6 | 0,0 | 1,1±0,02 |
| 2  **Activegel** 5000 mg / kg | 61,8±4,4* | 156,3±19,0 | 1,8±0,2 | 2,5±0,1 | 37,5±0,8 | 8,5±0,6 | 1,9±0,2 | 63,9±1,6 | 9,2±0,3 | 0,0 | 1,0±0,03 |
| 3  **Activegel** 2000 mg / kg | 64,8±6,5 | 151,5±17,0 | 2,2±0,1 | 2,5±0,1 | 38,3±3,4 | 8,6±0,3* | 2,5±0,2 | 62,9±1,3 | 8,2±0,3 | 0,0 | 1,2±0,03 |
| 4  **Activegel** 500 mg / kg | 62,3±2,4* | 141,0±14,3 | 1,8±0,1 | 2,4±0,1 | 38,8±2,0 | 8,9±0,5 | 2,4±0,1 | 60,1±2,0 | 8,0±0,6 | 0,0 | 1,1±0,03 |

Note: * statistically significant differences compared with the control group, p <0,05.

Table 2

**Evaluation of the effect of "Activegel" on the biochemical parameters of the peripheral blood of female rats 48 hours after 1 subcutaneous injection.**

| Group number | Biochemical parameters of peripheral blood of experimental animals | | | | | | | | | | |
| --- | --- | --- | --- | --- | --- | --- | --- | --- | --- | --- | --- |
|  | АLТ, u/l | АSТ, u/l | Total bilirubin, μmol / l | Calcium, mlmol / l | Creatinine,  μmol / l | Glucose, mlmol / l | Inorganic phosphorus, mmol / l | Total protein, g / l | Urea, mlmol / l | C-reactive protein, mlg / l | Ca +++, mlmol / l |
| 5  control | 58,0±6,9 | 129,3±12,4 | 1,8±0,1 | 2,5±0,1 | 39,0±1,1 | 8,9±0,9 | 1,6±0,1 | 66,2±1,8 | 10,6±0,4 | 0,0 | 1,1±0,02 |
| 6  **Activegel** 5000 mg / kg | 65,5±13,8 | 142,8±28,3 | 1,9±0,1 | 2,6±0,1 | 41,3±1,3 | 9,7±0,4 | 2,1±0,1* | 69,5±1,8 | 9,7±0,7 | 0,0 | 1,1±0,01 |
| 7  **Activegel** 2000 mg / kg | 54,3±3,8 | 131,5±7,2 | 1,6±0,1 | 2,4±0,1 | 39,0±0,9 | 7,2±1,0 | 2,0±0,1* | 65,3±2,9 | 10,5±0,3 | 0,0 | 1,1±0,03 |
| 8  **Activegel** 500 mg / kg | 57,0±3,2 | 138,8±6,6 | 1,5±0,2 | 2,4±0,1 | 43,5±2,0 | 8,4±0,5 | 2,2±0,1* | 62,5±1,5 | 9,0±0,3* | 0,0 | 1,1±0,04 |

Note: * statistically significant differences compared with the control group, p <0,05.

Table 3

**Evaluation of the effect of "Activegel" on the biochemical parameters of the peripheral blood of male rats 14 days after 1 subcutaneous injection.**

| Group number | Biochemical parameters of peripheral blood of experimental animals | | | | | | | | | | |
| --- | --- | --- | --- | --- | --- | --- | --- | --- | --- | --- | --- |
|  | АLТ, u/l | АSТ, u/l | Total bilirubin, μmol / l | Calcium, mlmol / l | Creatinine,  μmol / l | Glucose, mlmol / l | Inorganic phosphorus, mmol / l | Total protein, g / l | Urea, mlmol / l | C-reactive protein, mlg / l | Ca +++, mlmol / l |
| 9  control | 73,5±7,1 | 195,5±16,0 | 1,4±0,3 | 2,4±0,08 | 33,3±1,1 | 7,6±0,5 | 2,5±0,2 | 57,1±2,1 | 8,2±0,2 | 0,0 | 1,1±0,02 |
| 10  **Activegel** 5000 mg / kg | 72,8±3,8 | 160,8±13,9 | 1,8±0,2 | 2,5±0,03 | 33,8±1,5 | 9,6±0,3* | 2,4±0,1 | 58,3±1,5 | 8,2±0,2 | 0,0 | 1,2±0,01* |
| 11  **Activegel** 2000 mg / kg | 70,5±2,3 | 165,3±13,3 | 1,9±0,2* | 2,5±0,06 | 35,0±1,3 | 9,6±0,2* | 2,7±0,2 | 58,7±1,6 | 8,3±0,6 | 0,0 | 1,2±0,01* |
| 12  **Activegel**  500 mg / kg | 71,3±4,4 | 191,5±20,1 | 2,0±0,1* | 2,4±0,03 | 36,5±1,8 | 10,0±0,4* | 2,4±0,1 | 59,9±1,6 | 8,1±0,5 | 0,0 | 1,2±0,01 |

Note: * statistically significant differences compared with the control group, p <0,05.

Table 4

**Evaluation of the effect of "Activegel" on the biochemical parameters of the peripheral blood of female rats 14 days after 1 subcutaneous injection.**

| Group number | Biochemical parameters of peripheral blood of experimental animals | | | | | | | | | | |
| --- | --- | --- | --- | --- | --- | --- | --- | --- | --- | --- | --- |
|  | АLТ, u/l | АSТ, u/l | Total bilirubin, μmol / l | Calcium, mlmol / l | Creatinine,  μmol / l | Glucose, mlmol / l | Inorganic phosphorus, mmol / l | Total protein, g / l | Urea, mlmol / l | C-reactive protein, mlg / l | Ca +++, mlmol / l |
| 13  control | 52,8±4,5 | 126,5±7,1 | 1,7±0,2 | 2,5±0,1 | 41,5±4,8 | 8,0±0,2 | 1,5±0,2 | 66,6±2,8 | 9,6±0,4 | 0,0 | 1,1±0,01 |
| 14  **Activegel** 5000 mg / kg | 45,0±7,9 | 138,5±21,6 | 1,0±0,4 | 2,5±0,1 | 36,8±1,7 | 8,1±0,4 | 2,0±0,3 | 65,0±2,4 | 9,3±0,5 | 0,0 | 1,1±0,03 |
| 15  **Activegel** 2000 mg / kg | 54,5±9,8 | 135,3±7,8 | 1,6±0,3 | 2,6±0,03 | 36,8±1,3 | 7,5±1,0 | 1,6±0,1 | 67,9±0,8 | 9,5±0,8 | 0,0 | 1,2±0,01* |
| 16  **Activegel** 500 mg / kg | 49,0±2,9 | 133,5±14,9 | 1,8±0,2 | 2,4±0,1 | 38,5±1,5 | 6,6±0,4* | 2,2±0,2* | 63,3±4,0 | 9,1±0,6 | 0,0 | 1,1±0,04 |

Note: * statistically significant differences compared with the control group, p <0,05.
